# Discounting of Hyper-Palatable Foods Is Associated with Eating Motives and Binge Eating Behavior

**DOI:** 10.3390/nu17081356

**Published:** 2025-04-16

**Authors:** Joseph S. Bellitti, Alexa M. L’Insalata, Tera L. Fazzino

**Affiliations:** 1 Department of Psychology, University of Kansas, Lawrence, KS 66045, USA; 2 Cofrin Logan Center for Addiction Research and Treatment, University of Kansas, Lawrence, KS 66045, USA

**Keywords:** eating behaviors, binge eating, choice, motivation, food reward, loss of control

## Abstract

**Background**: High delay discounting (DD), or the tendency to prefer immediate rewards over larger delayed rewards, has been associated with health risk behaviors. This study examined the DD of hyper-palatable foods (HPFs) and money, and their associations with eating motives and binge eating behavior. **Methods**: An adult sample (N = 296) completed a DD task with single- and cross-commodity conditions with rewards of money and/or HPF (e.g., HPF now vs. HPF later; HPF now vs. money later). Regression models tested the association between DD, and eating motives and binge eating. **Results**: DD in the HPF now vs. money later condition was positively associated with the reward enhancement motive (β = 0.291; *p* = 0.008) and binge eating behavior (β = 0.041; *p* = 0.017). There were no other significant associations. **Conclusions**: Decisional impulsivity specific to HPFs (and not decisional impulsivity in general) may be associated with reward-motivated eating and binge eating behavior.

## 1. Introduction

Impulsivity is an umbrella term that encompasses various characteristics related to impulsive thoughts, behavior, and decisional processes [1]. Delay discounting (DD) describes the tendency to choose smaller immediate rewards over larger delayed rewards and is considered to represent decisional impulsivity [2]. A robust body of literature, including several meta-analyses [3,4] has demonstrated that elevated DD (i.e., a preference for smaller immediate rewards) is associated with greater substance use behavior. Most of the literature has examined the discounting of money, which has an explicit value across participants and can readily be compared across studies. Thus, most work has demonstrated that greater discounting of money is strongly associated with greater substance use behaviors.

More recently, advancements in the DD research domain have yielded more nuanced examinations of preferences for money relative to rewards that are most relevant to a disorder (e.g., alcohol for individuals with risky drinking behavior), termed cross-commodity DD. Importantly, cross-commodity DD paradigms can enhance the ecological validity of DD tasks by asking participants to choose between disorder-relevant rewards (e.g., alcohol) and a strong alternative reward (i.e., money), which may better parallel decisions made in the real world. Additionally, single-commodity DD tasks may facilitate simple explanations of decision-making behavior that in reality may be more nuanced [5]. For example, high DD in single-commodity tasks is typically interpreted as individuals having an inability to wait for a delayed rewards (because they typically choose the immediate reward in the task). However, an alternative interpretation is that individuals may disprefer the reward available at a delay (e.g., large quantities of a substance may be considered aversive), and therefore, individuals may tend to select the immediate reward. Individuals may be willing to wait for a disorder-specific reward under certain conditions; however, this cannot be elucidated from single-commodity tasks. Cross-commodity DD can overcome this limitation and can more clearly elucidate patterns of preference and decision-making by switching the immediate and delayed reward types across tasks (e.g., tasks of alcohol now money later or money now alcohol later). In doing so, researchers can distinguish whether individuals may prefer a reward when immediately available and whether they are willing to wait for their preferred reward at a delay because the alternative reward (e.g., money) in the task would be different from the target reward (e.g., alcohol). Thus, cross-commodity DD tasks help to more thoroughly elucidate choice patterns for different health-relevant rewards. In support of this premise, cross-commodity DD studies have found that individuals who engaged in greater substance use exhibited a preference for immediate rewards and were willing to wait for their substance of choice (e.g., alcohol and cannabis) when available at a delay [6,7]. Thus, cross-commodity DD tasks can reveal choice patterns and willingness to wait for a commodity that is aligned with an individual’s use behavior or disorder.

Binge eating behaviors have notable parallels with substance use, both in patterns of use and decisional processes that may lead to use [8,9]. Regarding use patterns, binge eating and substance use both occur on a dimensional scale from infrequent, occasional use of a substance/rewarding food to compulsive and excess use in binge drinking occasions or binge eating episodes. Binge eating behavior may range from passive overconsumption, which may occur when inattentively consuming highly rewarding foods, to loss of control eating, which is more characteristic of objective binge eating behavior identified in the eating disorder literature [10,11,12,13]. On this continuum, as has been observed with substances, individuals may be motivated to consume palatable foods for their acute rewarding effects and/or to cope with negative emotions during binge eating occasions [14,15,16,17,18]. Foods that are acutely rewarding to consume and are typically the target of binge eating occasions [14,16] are hyper-palatable foods (HPFs), which contain combinations of palatability-inducing nutrients (e.g., fat and sodium) at moderate to high levels that do not occur in nature [19,20,21,22]. HPFs are strong reinforcers similar to other psychoactive substances and are hypothesized to influence wanting and drive to consume them in a similar manner as psychoactive substances [21,23,24]. The incentive sensitization theory of motivational behavior, which was originally developed to describe substance use behavior [25] and was subsequently applied to food [26], posits that individuals initially may seek out HPFs due to the initial liking and acute rewarding effects of the foods [27,28]. Over time, repeated exposure to HPFs through binge eating may disrupt reward and motivation pathways in the brain and serve to dysregulate food reinforcement processes. Individuals may experience greater wanting or desire to consume HPFs and become hyper-sensitive to food cues in the environment, termed sensitization, leading to further HPF seeking and intake behavior [29,30,31]. Sensitization may lead to increased cravings and compulsive eating and drive further HPF intake during binge episodes.

Given that binge eating of HPFs shares similar reinforcement and sensitization patterns to substance use, it may be reasoned that decisional processes related to DD may also play a role in binge eating as they do in substance use behavior. For example, individuals with elevated DD may prefer the immediate rewarding effects of HPFs over delayed rewards such as long-term physical and mental health. This premise aligns with findings in the literature that demonstrated individuals who engaged in binge eating had higher instances of health conditions such as obesity [32,33] and type 2 diabetes [33,34], which may indicate individuals who binge eat may prefer immediate rewards from HPFs over larger delayed rewards such as healthy body weight. Therefore, binge eating may be associated with higher rates of DD, which would mirror the robust literature on DD for substance use [35].

Among preliminary studies of individuals who engage in binge eating behavior, discounting of money has been examined [36,37]; however, discounting of HPFs has not, which represents a significant gap in the literature. In the closest area of the literature to date, one prior study that used cross-commodity DD tasks revealed that individuals with excess dietary intake of HPFs and elevated HPF cravings tended to discount HPFs more steeply compared with a strong alternative reward (money), suggesting that discounting was specific to HPFs and not to rewards generally [38]. Thus, preliminary evidence suggests that the discounting of HPFs may be particularly relevant for individuals who engage in excess HPF intake behavior. In addition to preliminary evidence, there are known characteristics of binge eating that may be expected to increase DD for HPFs specifically (and not rewards generally). For example, binge eating often involves elements of waiting to consume HPFs [39]. For example, individuals who binge eat are typically constrained by their environment (e.g., work and social events), may be unable to engage in binge eating at a specific moment, and may wait until later, effectively engaging in excess HPF intake at a time delay. Additionally, binge eating behaviors are commonly preceded by dietary restraint and caloric restriction [40,41,42], which can lead to increased discounting of food due to physiological hunger [43,44] and urges [45,46,47,48]. Thus, research is needed to distinguish DD for HPFs specifically from a general inability to wait for rewards, which can be achieved via cross-commodity DD tasks, while accounting for factors like hunger to address potential dietary restriction.

While motives to consume HPFs (e.g., to experience their rewarding effects or to cope with negative emotions) and binge eating behavior may be most robust among clinical populations with binge-spectrum disorders, both occur on a continuum among the general population [49] and are implicated in a variety of physical and mental health consequences such as insulin resistance (pre-diabetes), obesity, mood disturbances, and anxiety [50,51,52,53]. However, no prior work has examined the role of DD in motives to consume HPFs and binge eating behavior among the general population; all prior work has focused on clinical populations. Understanding the potential role of DD in eating motives and binge eating on a dimensional scale in the general population is critical from a prevention perspective; if DD plays a role in binge eating behavior, which may have downstream physical and mental health consequences, early intervention approaches could be designed to target DD as a risk factor, to prevent future health consequences.

The purpose of the current study was to examine the associations between the DD of HPFs, and palatable eating motives and binge eating behavior among a general population sample, characterizing binge eating behavior on a dimensional scale. DD was examined using single- and cross-commodity discounting tasks with HPF and money to distinguish the DD of HPF relative to other rewards (e.g., money). We hypothesized that greater willingness to wait for HPFs when available at a delay and a relative preference for HPFs when available immediately would be associated with the endorsement of higher binge eating behavior and palatable eating motives (i.e., coping and reward enhancement).

## 2. Materials and Methods

### 2.1. Study Procedure

The Institutional Review Board at the host university approved this study. Amazon Mechanical Turk (MTurk) was used to collect study data using the following eligibility criteria: (1) 18–65 years old; (2) US residency; (3) MTurk quality approval rating of ≥99%; and (4) completed >1000 studies on MTurk. US residency was a necessary criterion to align with the study measures that used US foods and currency. The study surveys were released in six batches, which facilitated the recruitment of participants with differing availability in their schedules. Participants provided informed consent, completed surveys that took approximately 60 min to complete, and were compensated USD 4.50 for study completion. This study was not pre-registered.

### 2.2. Measures

#### 2.2.1. Delay Discounting Task

DD was measured using a computerized adjusting amount task that included both money (i.e., USD) and HPF commodities in single- and cross-commodity conditions. Participants selected their preferred HPF from a list of 12 options (Table 1), which were identified using a standardized definition in the literature [22]. The following single- and cross-commodity DD conditions were used in this study: (1) money now vs. money later; (2) HPF now vs. HPF later; (3) money now vs. HPF later; and (4) HPF now vs. money later. Participants completed each condition for commodity amounts that were smaller in magnitude and for commodity amounts that were larger in magnitude. Specifically, the smaller magnitude rewards were USD 10 or 4 servings of food, and the larger magnitude rewards were USD 100 or 40 servings of food. The HPF servings were derived using an exchange rate of USD 2.50 = 1 serving of food based on the market value for one serving of the food options presented to the participants (e.g., USD 2.50 per serving × 4 servings = USD 10 equivalent).

The DD conditions each contained multiple trials. Within each trial, participants would choose between a smaller reward available immediately and a larger reward available at a delay (e.g., would you prefer USD 5 now or USD 10 in a day?). The initial values for immediate rewards in each trial were calculated as 50% of the delayed reward. For example, if the delayed reward was 4 servings of HPF, then the corresponding immediate reward would start at 2 servings of HPF. The immediate reward in the subsequent trial was dependent upon the participant’s previous response. Specifically, if the participant selected the immediate reward, the immediate reward in the next trial was adjusted down by 50% of the prior adjustment (e.g., USD 2.50). Contrastingly, if the delayed reward was selected, the immediate reward in the next trial was adjusted up by 50% of the prior adjustment (e.g., USD 7.50) [54]. This process occurred over six trials to identify each participant’s indifference point (i.e., their discounted value of the delayed outcome). These indifference points were collected over five delay periods (1 day, 1 week, 1 month, 6 months, and 1 year), and each was subsequently used to calculate a k value for analyses [54]. In total, the participants completed 240 trials.

#### 2.2.2. Palatable Eating Motives Scale

Motives to consume HPFs were assessed via the Palatable Eating Motives Scale-Revised (PEMS) [49]. This 19-item measure assesses four motives for consuming HPFs in the absence of physiological hunger. Coping and reward enhancement motive scores were used in the analyses, in alignment with the study focus. The coping motive measures the tendency to eat tasty foods to forget about worry, to help with negative mood, or to manage problems. The reward enhancement motive assesses the tendency to eat tasty foods for rewarding or pleasant feelings induced by the food. Participants reported how frequently they consumed tasty foods in accordance with each motive using a 5-point Likert-scale (1 = never/almost never to 5 = almost always/always). The mean response of the scale items was calculated as coping and reward enhancement motives for the analyses. Research has demonstrated that the PEMS has good convergent validity with similar measures of eating behavior (e.g., Yale Food Addiction Scale; Binge Eating Scale) [49] as well as discriminant validity with distinct measures of motivational behavior (i.e., Sensitivity to Punishment; Reward Questionnaire) [49]. The reliability for the coping and reward enhancement subscales used in the current study was good (reward enhancement α = 0.82; coping α = 0.86).

#### 2.2.3. Binge Eating Behavior

The Eating Pathology Symptom Inventory (EPSI) [55] was used to assess binge eating behavior. The EPSI is a self-report measure designed to assess eight dimensions of eating pathology and has demonstrated robust psychometric properties in both clinical and non-clinical community samples [55]. For the present study, the binge eating subscale was used. The binge eating scale conceptualizes binge eating behavior on a continuum and is appropriate for use in healthy samples without eating disorders and in samples with eating disorders. The scale contains eight items that assess binge eating, characterized from passive overeating to loss of control eating symptoms over the past 4 weeks. The items were assessed using a 5-point Likert scale (0 = never to 4 = very often). The binge eating scores were calculated using a sum. The binge eating subscale of the EPSI has yielded adequate test–rest reliability among clinical, community, and college samples (α = 0.71) [55,56]. The binge eating scale has also evidenced strong internal consistency (α = 0.85) [55,57]. Furthermore, the binge eating scale has evidence of convergent validity and has been correlated with other measures of eating pathology such as the Eating Disorder Examination Questionnaire [58]. The alpha reliability coefficient was good for the present study (α = 0.80).

#### 2.2.4. Hunger

Previous studies have suggested that physiological hunger may increase the discounting of food [43,44]. Additionally, hunger may be considered as a proxy for dietary restriction given that greater restriction of calories may yield greater hunger [59]. Therefore, hunger was measured in the current study and accounted for in the analyses. Hunger was measured using a 100 mm visual analog scale (VAS). This scale asked participants to rate their current hunger level on a range from 0 (i.e., not hungry at all) to 100 (i.e., very hungry).

### 2.3. Data Analytic Plan

#### 2.3.1. Delay Discounting Parameter Calculation

For the present study, the DD rate was calculated using Mazur’s hyperbolic function, which indicates, V = A/1 + kD [60]. For this formula, V is the indifference point, A is the commodity value, D is the delay period (i.e., number of days), and k is the estimated participant DD rate. The K values for the present study were positively skewed. Thus, a logarithmic transformation of k values was completed as is typical for parametric analyses [61]. Higher ln(k) values reflect a higher DD rate (i.e., preference for smaller immediate rewards) relative to lower ln(k) values. It is typical for studies that measure DD rate to include figures that assess the line of best fit by displaying the median indifference points as a function of the time delay using Mazur’s hyperbolic equation [60]. However, the present study does not include these figures as these figures have been published previously [38,62] for each condition at each magnitude.

#### 2.3.2. Statistical Analyses

R software (Version 4.4.2) was used for all statistical analyses, and graphing techniques were used to investigate the model assumptions visually. There were no substantive deviations from normality observed in the Q-Q plots, and the assumptions for linear regression models were met. To test whether greater palatable eating motives (i.e., coping and reward enhancement motive) were associated with a greater willingness to wait for HPFs when available at a delay and a relative preference for HPFs when available immediately, single- and cross-commodity DD ln(k) values were used in six multiple regression models. Specifically, to test the association between the discounting of HPFs in single-commodity conditions with palatable eating motives, two multiple linear regression models were constructed with the ln(k) value from the single-commodity condition (i.e., HPF now vs. HPF later) as the independent variable and each PEMS score as the outcome. Next, to test the association between the discounting of HPF in cross-commodity conditions, four linear regression models were constructed with ln(k) values from the money now vs. HPF later and HPF now vs. money later conditions as the independent variable and each PEMS score as the outcome. For the binge eating outcomes, model construction mirrored those for palatable eating motives described above, except that EPSI binge eating score was used as the outcome. Covariates with potential theoretical and statistical relevance (e.g., hunger, income, and BMI) were tested for inclusion in the models. However, hunger rating was the only covariate that was meaningfully associated with the predictor and outcome variables and accounted for substantive variance in the models. Therefore, hunger was included as a covariate in all analytic models. Additionally, single-commodity money conditions for each outcome were calculated and included for comparison as is typical within the DD literature.

### 2.4. Data Quality Criteria and Missing Data

We examined non-systematic responding to ensure data quality and orderliness. In alignment with prior studies conducted with this dataset, Johnson and Bickel’s criteria [63] were applied to the data, resulting in the removal of 4.1% of participants in the small-magnitude analyses (*n* = 12) and 3.7% of participants in the large-magnitude analyses (*n* = 11). Please see prior publications [38,62] for more details. Thus, a sample of N = 296 was available and had systematic discounting data to include in the analyses. Additionally, several participants had missing data on the survey measures (i.e., PEMS and EPSI) and were excluded from the relevant analyses. For analyses using the PEMS coping scale, the final sample was n = 279 in the small-magnitude analyses and n = 281 in the large-magnitude analyses. For analyses using the PEMS reward enhancement scale, the final sample was n = 278 in the small-magnitude analyses and n = 280 in the large-magnitude analyses. For analyses using the EPSI, the final sample was n = 277 in the small-magnitude analyses and n = 279 in the large-magnitude analyses.

## 3. Results

### 3.1. Participants

**Figure 1:**
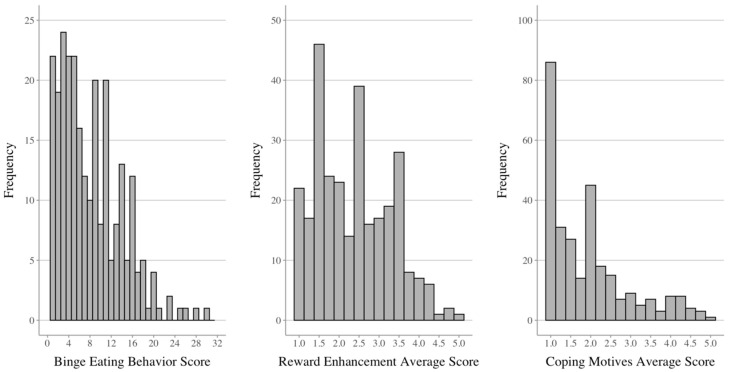
Distributions of dependent variables.

Sample characteristics (N = 296) and descriptive statistics for the outcomes are provided in Table 2. Nearly three-quarters of the sample identified as White/Non-Hispanic and reported employment (i.e., full or part-time). Regarding outcomes, the mean binge eating behavior score was 7.4 (SD = 6.0; R = 0–30) out of the possible 32 points, and participants endorsed the full range of responses on the measure (as depicted in the Figure 1 histogram). The reward enhancement eating and coping motives were an average of 2.4 points (SD = 0.9; R = 0–5) and 1.9 points (SD = 1.0; R = 0–5), respectively, and participants collectively endorsed the full range of the eating motives scales (Figure 1).

### 3.2. Linear Regression Analyses

#### 3.2.1. Eating Motives Analyses

**Figure 2:**
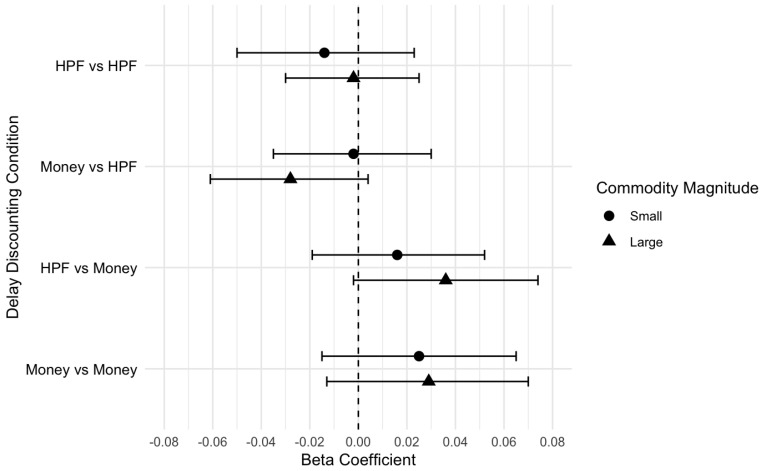
Effect size plot for coping motive outcomes (small and large magnitude).

**Figure 3:**
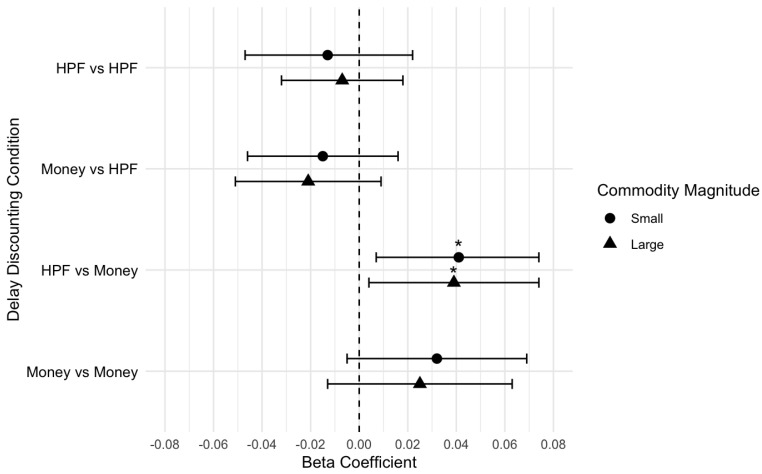
Effect size plot for reward enhancement motive outcomes (small and large magnitude). * *p* < 0.05.

There were no significant associations between DD and coping motive endorsement for single- and cross-commodity DD conditions, when controlling for hunger. This pattern of findings was consistent across small (Table 3) and large (Table 4) magnitudes for the coping motive. In contrast, there was a significant association between DD and reward enhancement motive endorsement in the cross-commodity condition of HPF now vs. money later, when controlling for hunger. Specifically, for every one unit increase in ln(k) value when choosing HPF now over money later, reward enhancement motive was 0.04 units higher (Table 5). This suggested, after controlling for hunger, that individuals who preferred HPFs immediately over delayed money tended to endorse higher reward enhancement. DD was not significantly associated with the other single- or cross-commodity conditions. This pattern of findings was identical across the small- and large-magnitude results (Table 6). Visual displays of the effect size plots are provided in Figure 2 for coping and Figure 3 for reward enhancement (Figure 3).

#### 3.2.2. Binge Eating

**Figure 4:**
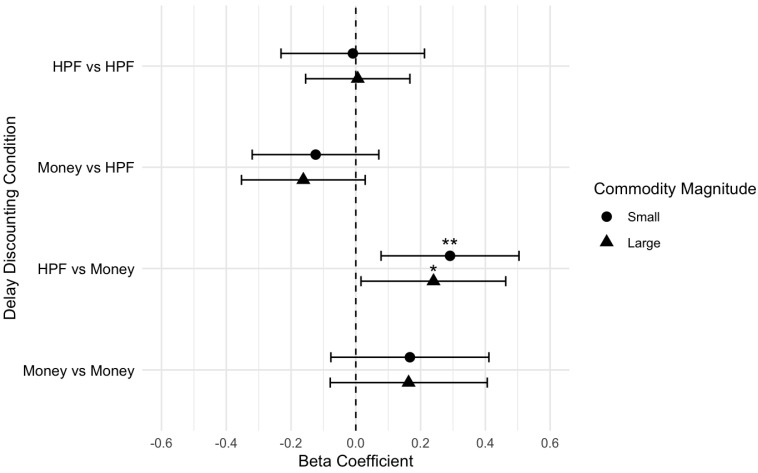
Effect size plot for binge eating behavior outcomes (small and large magnitudes). ** *p* < 0.01; * *p* < 0.05.

The results indicated there was a significant association between binge eating behavior and DD in the cross-commodity condition of HPF now vs. money later, when controlling for hunger. Specifically, for every one unit increase in ln(k) value when choosing HPFs now over money later, the binge eating score was 0.29 units higher (Table 7). This suggested that individuals who preferred HPFs immediately over delayed money tended to engage in greater binge eating behavior, even when controlling for hunger. Binge eating behavior was not significantly associated with the other single- or cross-commodity conditions (Table 7). This pattern of findings was similar across the small- and large-magnitude results (Table 8), and Figure 4 visually depicts the effect sizes.

## 4. Discussion

This study examined the role of delay discounting (DD) in palatable eating motives and binge eating behavior, characterized on a continuum in a general population sample. Overall, the findings demonstrated that the tendency to choose HPFs now over a strong alternative reward (money) was positively associated with binge eating behavior and the motive to consume HPFs for their rewarding effects. Notably, the findings were specific to the discounting of HPFs and were not observed in other discounting conditions with money as the reward. Thus, the findings suggested that discounting specific to HPFs, and not the tendency to discount delayed rewards more generally, may play a role in reward-motivated eating and binge eating behavior. The effects were present in both small- and large-magnitude DD conditions, which indicated the stability of the findings. The observed effect sizes were small to moderate and were consistent with the prior literature on the DD of HPFs for HPF-intake related outcomes [38] and the broader DD substance use literature [3]. Considered collectively, our findings and the robust prior literature suggests that DD is a relevant and consistent factor in decision-making related to health behaviors [3,4]. There were no other significant associations between the single- and other cross-commodity conditions, and eating to cope or binge eating behavior. Thus, our findings overall suggest that individuals among the general population who exhibit decisional impulsivity specific to HPFs may endorse greater motivation to consume HPFs for their rewarding effects and may engage in greater binge eating behavior.

The current study was the first to examine the role of discounting in palatable eating motives and found that elevated DD was associated with the motive to consume HPFs for their rewarding effects (i.e., reward enhancement motive). The effects were small in magnitude but were replicated across low- and high-magnitude DD conditions, suggesting the effect may be reliable and worth further examination. Considered in the context of theoretical support, the reward enhancement motive may be viewed as reflecting wanting or drive to consume HPFs, which occurs via sensitization [27]. DD for HPFs may facilitate acting on strong wanting and drives to consume HPFs, particularly in the presence of HPF cues (e.g., advertisements and signs) in the environment [28,31]. Furthermore, with repeated HPF consumption over time, HPFs may have impacts on areas of the brain involved in self-regulation [29,64,65], which may exacerbate existing discounting tendencies and facilitate further HPF seeking and intake. Nevertheless, due to the preliminary nature of these findings, future research is needed to examine these processes longitudinally.

The null findings from the coping analyses suggest that decisional impulsivity may not play a major role in coping-motivated eating. The results were surprising given that eating to cope with negative emotions has been a robust and reliable correlate with excess HPF intake and disordered eating behavior in the literature [66]. It may be that eating to cope is more of a planned experience and not driven by decisional impulsivity. However, if this was the case, we would have expected to observe a positive correlation between willingness to wait for HPFs later in the DD tasks and coping motive endorsement, although we did not. In the broader literature, specific types of impulsivity (e.g., reward sensitivity and attentional impulsivity) have been associated with HPF intake and related outcomes [67,68,69,70]. Therefore, more work is needed to understand whether and to what degree various types of impulsivity may be involved in coping-motivated eating.

Our findings extend the existing literature on DD for binge eating, which to date has focused on the DD of money using single-commodity tasks [36]. Given that components of the binge eating process may be expected to elevate discounting of HPF specifically, our methodological advancement in the use of cross-commodity DD tasks that assessed preferences for HPF relative to money represents a particular strength of this study. Furthermore, our use of a binge eating measure that was appropriate for healthy (non-clinical) populations facilitated a dimensional characterization of binge eating behavior. The participants utilized most of the binge eating behavior scale, which provided high variability in their responses and likely increased our ability to detect associations with discounting. Our resulting findings were nuanced and aligned with the premise that binge eating behavior may be related to decisional impulsivity specific to HPFs (and not decisional impulsivity more broadly). Our findings are consistent with one prior study that used a cross-commodity DD task and found that the tendency to choose HPFs immediately over delayed money was associated with the excess dietary intake of HPFs, craving for HPFs, and body mass index among a general population sample [38]. Overall, examining the cross-commodity DD of HPFs and money may have utility in characterizing the role of decisional impulsivity in excess HPF intake behaviors and, if our findings were to be replicated, may suggest that decisional impulsivity specific to HPFs may play a role in binge eating behavior.

In contrast to our hypotheses, we did not find that individuals who engaged in binge eating behavior were willing to wait for HPFs when available at a delay. While this was the first study to test this premise with binge eating, prior work in the substance use literature has reported that individuals with risky substance use were willing to wait for their substance of choice, assessed using cross-commodity discounting paradigms [5,6,7]. It may be that individuals with high decisional impulsivity toward HPFs are not willing to wait for HPFs, which may represent a distinction from substance use behavior. Another consideration may be that HPFs saturate the US food environment (69% of available foods as of 2018) [71] and are readily available in a manner distinct from other substances (e.g., alcohol and opioids). In this regard, the DD task may have had limited ecological validity for participants and may be reflected in the null findings.

Another consideration is that this study focused on one type of impulsivity (i.e., decisional impulsivity) and did not address other established eating constructs, such as emotional eating, which could have influenced the findings. For example, another aspect of impulsivity that may be relevant for our findings is urgency, defined as acting rashly in response to strong emotions. Urgency may be considered to reflect emotional eating, as addressed in the disordered eating literature, which involves eating in response to distress and negative emotions [72]. In our study, urgency and emotional eating would be most closely reflected in the coping motive, which addressed eating to cope with stress and negative emotions [49,73,74,75]. If urgency/emotional eating had a substantial role in the findings, we would have expected to observe significant associations across both coping and reward enhancement motives with DD of HPFs. However, there were no significant correlations between coping motive endorsement and the DD of HPFs (or money); rather, significant associations were observed only between reward enhancement and the DD of HPFs. Thus, our findings do not support the premise that urgency or emotional eating may explain our observed results. However, given the robust associations observed in the prior literature regarding emotional eating, urgency, and disordered eating, future work is needed to replicate and extend these findings.

This study had several limitations. First, the sample was a convenience sample primarily comprising White, college-educated individuals, which may limit generalizability to the broader US population. Additionally, this sample was drawn from a crowdsourcing platform, and as such, there may be additional limitations to generalizability; the individuals may have had familiarity with experimental and behavioral tasks and had awareness of the attention check procedures commonly employed in crowdsourced surveys to ensure data quality [76]. Furthermore, the participants were aware that they were completing a study on food choices, which may have introduced demand characteristics. It is possible that implicit measures (e.g., implicit association tests) may have additive utility in examining the relationship between decisional impulsivity, and binge eating and eating motives in such contexts. Third, this study used hypothetical rather than real rewards in the DD task [77], which may impact the perception of risk and decision-making during the task [78]. Specifically, using hypothetical rewards may have introduced response bias as participants’ choices may have been different if presented with real rewards (e.g., actual food and money). Nevertheless, research specifically comparing hypothetical and real DD data that use food rewards has indicated they may closely align [44]. Another important limitation is this study did not include a validated dietary restraint measure. Given that dieting practices like dietary restraint and caloric restriction have been found to be a predictor of binge eating [40,41,42], it is important to account for how such behaviors may influence their discounting. We attempted to address this limitation by including hunger as a covariate in all analyses to serve as a proxy for measuring dietary restriction, which is supported by prior work [14]. However, future research should include more standardized measures of dietary restraint to further refine the analyses. Finally, our study was cross-sectional, and causal inferences cannot be drawn from our findings. For example, we were unable to test whether greater decisional impulsivity for HPFs may lead to increased risk for binge eating behavior over time. Future work should investigate these relationships, leveraging a longitudinal study design that can further establish causal relationships.

## 5. Conclusions

Our findings overall provide initial evidence that among a general population sample, decisional impulsivity specific to HPFs may play a role in reward-motivated eating and binge eating behavior. Our methodological advancement that employed cross-commodity DD was useful in delineating how choices between HPF and alternative rewards may relate to eating motives and behavior. Our findings may be considered in the context of eating disorder prevention efforts and suggest that individuals who exhibit decisional impulsivity specific to HPFs (rather than decisional impulsivity broadly) may be at elevated risk for binge eating behavior and reward-motivated eating and may be candidates for prevention interventions. For example, future-oriented approaches (e.g., episodic future thinking) have shown evidence for reducing individual DD rates and may be helpful for prevention efforts as well [79]. Additionally, researchers may consider addressing decisional impulsivity toward HPFs, which may help address reward-driven eating and binge eating behavior and possibly prevent the development of disordered eating and related health consequences.

## Figures and Tables

**Table 1:** HPF options for delay discounting task.

Hot dog
Pizza
Cheeseburger
Potato chips
Reese’s Puffs Cereal
Ice cream
Snickers
M&Ms
French fries
Saltine crackers
Pretzels
White bread or roll

Note. Hyper-palatable food = HPF.

**Table 2:** Sample characteristics (N = 296).

Variable	Mean (SD) or N (%)
**Gender**	
Man	170 (57.4)
Woman	125 (42.2)
Transgender	1 (<1)
**Race/Ethnicity**	
White/Non-Hispanic	215 (72.6)
White/Hispanic	14 (4.7)
Black/Non-Hispanic	23 (7.8)
Asian/Non-Hispanic	28 (9.5)
Native American	3 (1.0)
Multiracial/ethnicity	13 (4.4)
**Age**	38.27 (11.01)
**Education**	
<High School GED	1 (<1)
High School GED or Equivalent	31 (10.5)
Some college, no degree	58 (19.6)
Post-secondary degree	131 (44.3)
Graduate/Professional degree	47 (15.9)
Not Reported	28 (9.5)
**Income**	
<20 k	29 (9.8)
20 k–49,999	81 (27.4)
50 k–99,999	112 (37.8)
100 k+	46 (15.5)
Not Reported	28 (9.5)
**Employment**	
Full/Part-time	216 (72.9)
Unemployed/Disabled	49 (16.6)
Not Reported	31 (10.5)
**Palatable Eating Motives Scale**	
Reward Enhancement	2.37 (0.93)
Coping	1.92 (1.00)
**Eating Pathology Symptoms Inventory**	
Binge Eating	7.35 (6.01)

Note. The demographic data were obtained from our survey. For race/ethnicity, the participants were allowed to select more than one option.

**Table 3:** Linear regression with small-magnitude discounting conditions and PEMS Coping Scale (N = 279).

Condition	Estimate (95% CI)	t Value	SE	*p* Value
HPF vs. HPF	−0.014 (−0.050–0.023)	−0.749	0.02	0.454
Money vs. HPF	−0.002 (−0.035–0.030)	−0.144	0.02	0.885
HPF vs. Money	0.016 (−0.019–0.052)	0.903	0.02	0.368
Money vs. Money	0.025 (−0.015–0.065)	1.246	0.02	0.214

Note: Hunger was included as a covariate in all models. CI = confidence interval; HPF = hyper-palatable food; PEMS = Palatable Eating Motives Scale.

**Table 4:** Linear regression with large-magnitude discounting conditions and PEMS Coping Scale (N = 281).

Condition	Estimate (95% CI)	t Value	SE	*p* Value
HPF vs. HPF	−0.002 (−0.030–0.025)	−0.154	0.01	0.878
Money vs. HPF	−0.028 (−0.061–0.004)	−1.717	0.02	0.087
HPF vs. Money	0.036 (−0.002–0.074)	1.849	0.02	0.066
Money vs. Money	0.029 (−0.013–0.070)	1.369	0.02	0.172

Note: Hunger was included as a covariate in all models. CI = confidence interval; HPF = hyper-palatable food; PEMS = Palatable Eating Motives Scale.

**Table 5:** Linear regression with small-magnitude discounting conditions and PEMS Reward Enhancement Scale (N = 278).

Condition	Estimate (95% CI)	t Value	SE	*p* Value
HPF vs. HPF	−0.013 (−0.047–0.022)	−0.738	0.02	0.461
Money vs. HPF	−0.015 (−0.046–0.016)	−0.978	0.02	0.329
HPF vs. Money	0.041 (0.007–0.074)	2.409	0.02	0.017
Money vs. Money	0.032 (−0.005–0.069)	1.692	0.02	0.092

Note: Hunger was included as a covariate in all models. CI = confidence interval; HPF = hyper-palatable food; PEMS = Palatable Eating Motives Scale.

**Table 6:** Linear regression with large-magnitude discounting conditions and PEMS Reward Enhancement Scale (N = 280).

Condition	Estimate (95% CI)	t Value	SE	*p* Value
HPF vs. HPF	−0.007 (−0.032–0.018)	−0.539	0.01	0.590
Money vs. HPF	−0.021 (−0.051–0.009)	−1.364	0.02	0.174
HPF vs. Money	0.039 (0.004–0.074)	2.196	0.02	0.029
Money vs. Money	0.025 (−0.013–0.063)	1.305	0.02	0.193

Note: Hunger was included as a covariate in all models. CI = confidence interval; HPF = hyper-palatable food; PEMS = Palatable Eating Motives Scale.

**Table 7:** Linear regression with small-magnitude discounting conditions and EPSI Binge Eating Scale (N = 277).

Condition	Estimate (95% CI)	t Value	SE	*p* Value
HPF vs. HPF	−0.009 (−0.231–0.212)	−0.083	0.11	0.934
Money vs. HPF	−0.124 (−0.320–0.071)	−1.251	0.10	0.212
HPF vs. Money	0.291 (0.078–0.504)	2.688	0.11	0.008
Money vs. Money	0.167 (−0.077–0.411)	1.351	0.12	0.178

Note: Hunger was included as a covariate in all models. CI = confidence interval; HPF = hyper-palatable food; EPSI = Eating Pathology Symptom Inventory.

**Table 8:** Linear regression with large-magnitude discounting conditions and EPSI Binge Eating Scale (N = 279).

Condition	Estimate (95% CI)	t Value	SE	*p* Value
HPF vs. HPF	0.006 (−0.155–0.167)	0.073	0.08	0.942
Money vs. HPF	−0.162 (−0.353–0.029)	−1.670	0.10	0.096
HPF vs. Money	0.240 (0.016–0.463)	2.112	0.11	0.036
Money vs. Money	0.163 (−0.079–0.406)	1.328	0.12	0.185

Note: Hunger was included as a covariate in all models. CI = confidence interval; HPF = hyper-palatable food; EPSI = Eating Pathology Symptom Inventory.

## Data Availability

The data will be provided upon reasonable request to the PI and approval of the Institution Review Board. The data are not publicly available due to privacy and ethical restrictions.
